# Systemic Oxidative Stress Biomarkers in Chronic Periodontitis: A Meta-Analysis

**DOI:** 10.1155/2014/931083

**Published:** 2014-11-16

**Authors:** Zhiqiang Liu, Yan Liu, Yiqing Song, Xi Zhang, Songlin Wang, Zuomin Wang

**Affiliations:** ^1^Department of Stomatology, Beijing ChaoYang Hospital Affiliated to Capital Medical University, Beijing 100020, China; ^2^Beijing Key Laboratory of Tooth Regeneration and Function Reconstruction, Capital Medical University School of Stomatology, Beijing 100050, China; ^3^Department of Epidemiology, Indiana University Richard M. Fairbanks School of Public Health, Indianapolis, IN 46202, USA; ^4^Institute of Vascular Medicine, Peking University Third Hospital, Beijing 100191, China; ^5^Laboratory of Gene Therapy and Tooth Regeneration, Beijing Key Laboratory of Tooth Regeneration and Function Reconstruction, Capital Medical University School of Stomatology, Beijing 100050, China

## Abstract

Oxidative stress biomarkers have been observed in peripheral blood of chronic periodontitis patients; however, their associations with periodontitis were not consistent. This meta-analysis was performed to clarify the associations between chronic periodontitis and oxidative biomarkers in systemic circulation. Electronic searches of PubMed and Embase databases were performed until October 2014 and articles were selected to meet inclusion criteria. Data of oxidative biomarkers levels in peripheral blood of periodontitis patients and periodontal healthy controls were extracted to calculate standardized mean differences (SMDs) and 95% confidence intervals (CIs) by using random-effects model. Of 31 eligible articles, 16 articles with available data were included in meta-analysis. Our results showed that periodontitis patients had significantly lower levels of total antioxidant capacity (SMD = −2.02; 95% CI: −3.08, −0.96; *P* = 0.000) and higher levels of malondialdehyde (SMD = 0.99; 95% CI: 0.12, 1.86; *P* = 0.026) and nitric oxide (SMD = 4.98; 95% CI: 2.33, 7.63; *P* = 0.000) than periodontal healthy control. Superoxide dismutase levels between two groups were not significantly different (SMD = −1.72; 95% CI: −3.50, 0.07; *P* = 0.059). In conclusion, our meta-analysis showed that chronic periodontitis is significantly associated with circulating levels of three oxidative stress biomarkers, indicating a role of chronic periodontitis in systemic diseases.

## 1. Introduction

Chronic periodontitis, characterized by inflammation and destruction of periodontal supporting tissues, is one of the most common oral diseases worldwide. Over 47% of American people had chronic periodontitis [1], and the prevalence is even higher in developing countries [2]. Chronic periodontitis is initially caused by various hyperresponsive and destructive products of immune response stimulated by microbial plaque around the gingival margin.

In the pathogenesis of periodontitis, polymorphonuclear leukocytes (PMN) act as the primary mediators of the host response against proliferating periodontal pathogenic microorganisms. Activated PMN produce a large amount of reactive oxygen species (ROS) and result in destruction of periodontal tissues [3, 4]. There is some suggestive evidence that periodontal inflammation might be associated with systemic oxidative stress. Recently, abundant evidence has shown that periodontal diseases were highly associated with several inflammation-related systemic diseases, such as chronic respiratory diseases [5], cardiovascular disease [6], and diabetes mellitus [7]. Oxidative stress plays an important role in the pathogenesis of these diseases [8–11]. It has been hypothesized that oxidative stress arising from periodontal lesions may be an important cause of systemic inflammation. Some but not all epidemiological studies have shown that biomarkers levels of oxidative stress in the peripheral blood of periodontitis patients were different from periodontal healthy subjects [12–14]. However, different levels of oxidative stress biomarkers had been detected in peripheral blood of chronic periodontitis patients in different studies, and also their findings were not consistent.

To test the hypothesis that chronic periodontitis is associated with systemic oxidative stress, we therefore carried out a meta-analysis of all published relevant cross-sectional, case control, and intervention studies to provide a comprehensive and quantitative synthesis of accumulative evidence.

## 2. Methods

### 2.1. Search Strategy

A literature search was conducted using the PubMed and Embase databases update to October 2014. The search strategy was as follows: (1) keywords “oxidat^*^,” “antioxidat^*^,” “oxidant^*^,” “antioxidant^*^,” “redox,” “reactive oxygen species,” “ROS,” and common used oxidative stress biomarkers keywords “total antioxidant capacity,” “TAOC,” “total oxidant status,” “TOS,” “total antioxidant status,” “TAS,” “oxidative stress index,” “OSI,” “nitric oxide,” “malondialdehyde,” “MDA,” “superoxide dismutase,” “SOD,” “reactive oxygen metabolites,” “ROM,” “glutathione,” “GSH,” “glutathione peroxidase,” “GPx,” “8-hydroxy-deoxyguanosine,” “8-OHdG,” and “catalase” were connected by Boolean operator “OR”; (2) keywords “serum,” “plasma,” and “blood” were connected by Boolean operator “OR”; (3) the above search results and the keyword “periodont^*^” were connected by Boolean operator “AND”. All keywords were restricted in title or abstract without the language limitation.

### 2.2. Study Selection

Two reviewers scanned the titles and abstracts of all above searched articles independently to look for the relevant studies reporting the biomarkers levels of oxidative stress in periodontitis patients and healthy controls. The inclusion criteria were as follows: (1) study subjects were human adults; (2) 7.both periodontitis patients group and periodontal healthy control group were included in the study; (3) at least one oxidative stress biomarker was measured in peripheral blood samples; (4) language was English. If any of above criteria could not be identified only through title and abstract, full text of the article was reviewed.

Full texts of all above included articles were then reviewed for a further selection. The exclusion criteria were as follows: (1) smokers and/or subjects with systemic diseases were not excluded from periodontitis group and/or periodontal healthy control group; (2) biomarkers levels were only shown by histogram with no detailed data; (3) if two articles have the same study population, only the latest published article was selected; (4) oxidative biomarkers were only measured in red blood cells or lymphocytes. Any disagreements were discussed for a consensus.

### 2.3. Data Extraction

Data were extracted by two reviewers independently. Mean ± SD, median (min–max), or median (25%–75%) of peripheral blood oxidative stress biomarkers in periodontitis patients and periodontal healthy control groups was extracted. If it was a clinical intervention study, we only extracted the baseline data before periodontal treatment. When there were more than two groups in one study, we only focused on the data of systemic healthy periodontitis patients and healthy control.

### 2.4. Quality Assessment

Newcastle-Ottawa Quality Assessment Scale (NOS) (http://www.ohri.ca/programs/clinical_epidemiology/oxford.asp) was used to assess the methodological quality of all the included nonrandomized studies. The NOS for case control studies includes three domains (selection, comparability, and outcomes) and eight items. A study can be awarded a maximum of one star for each numbered item within the selection and exposure categories. A maximum of two stars can be given for comparability. Clinical intervention studies included in this study were assessed with the same scale as case control studies, because only baseline data were focused on and extracted in our analysis. Two reviewers worked independently, and discrepancies were resolved by discussion.

### 2.5. Statistical Analyses

Meta-analyses were conducted to summarize the differences levels of oxidative stress biomarkers between periodontitis patients and healthy controls, if there were 3 more studies reporting the same biomarker measurement and the biomarker levels were expressed by mean ± SD.

Since the included studies used different assay methods and units for the same biomarker, we calculated the standardized mean difference (SMD) and 95% confidence interval (CI) as a summary statistic in meta-analysis for the difference of the levels of each biomarker between periodontitis patients and periodontal healthy controls. SMD is the mean divided by the standard deviation of a difference in each biomarker between patients and controls and can be seen as the mean difference that would have been obtained if all data were transformed to a scale where the standard deviation within groups was equal to 1.0.

Heterogeneity among studies was assessed by *Q* test, which indicated a significant heterogeneity if *P* value <0.05. We also quantified the extent of heterogeneity with the *I* ^2^ value, where the percentages of *I* ^2^ 25–50%, 50–75%, and >75% indicate low, medium, and high heterogeneity, respectively. A random-effects model was used to calculate SMD when there was a significant heterogeneity; otherwise a fixed-effects model was used. Statistical analyses were carried out using the Stata statistical software version 12.0 (Stata, College Station, TX, USA).

## 3. Results

### 3.1. Study Selection and Characteristics

**Figure 1:**
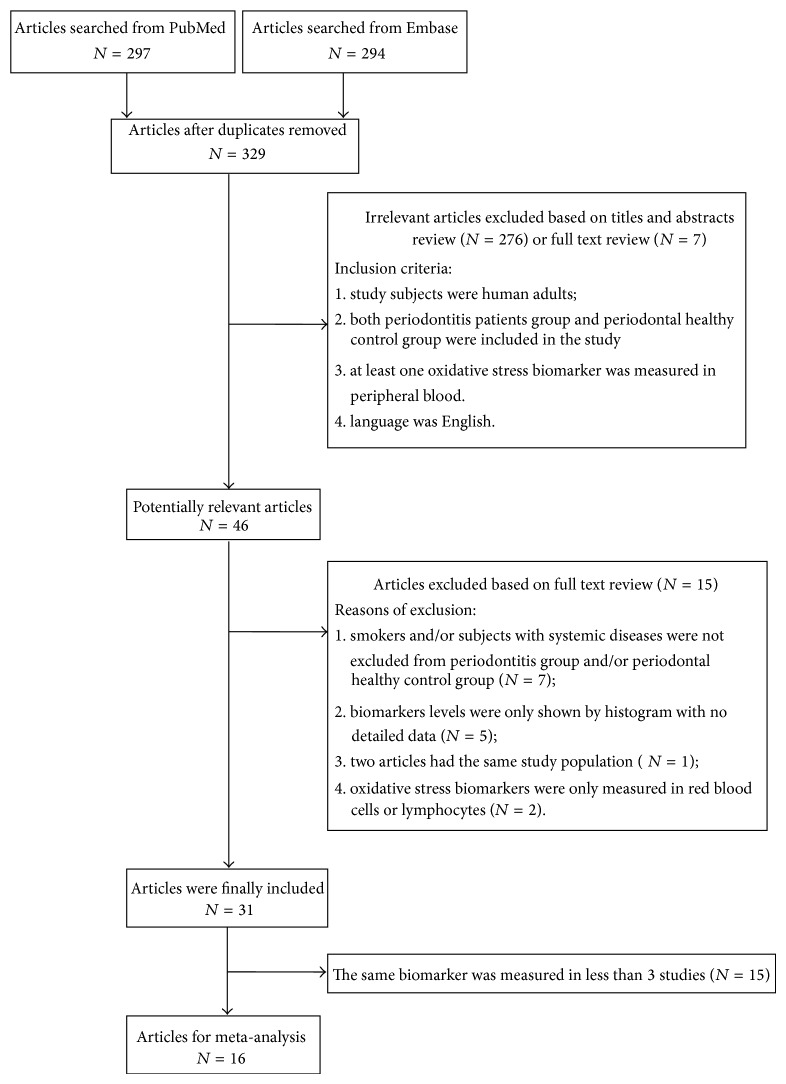
Flow chart of the process of study selection.

A total of 297 and 294 articles were separately screened based on electronic search from PubMed and Embase database. 329 articles were left after duplicates were removed. Only 31 relevant articles published from December 2002 to September 2014, including 21 cross-sectional or case-control design studies and 10 clinical intervention studies, were finally selected after a title or abstract review and a full text review according to the exclusion and inclusion criteria. At last, 16 articles were included in meta-analysis. The process of study selection and the specific information of these studies were depicted in Figure 1 and basic characteristics of all included studies were summarized in Table 1.

### 3.2. Quality Assessment

The results of quality assessment with NOS were shown in Supplemental Table  1 available online at http://dx.doi.org/10.1155/2014/931083. All included studies had middle or high quality. Seven studies were given 6 stars, 20 studies were given 7 stars, and 4 studies were given 8 stars. Almost all studies met the NOS criteria for case definition and had good representativeness of cases. However, for selection of controls, almost all studies derived from the same population as the cases, but hospital controls were used. In all studies, controls had good periodontal status when they were included in the studies, but these studies did not explicitly state that controls had no history of periodontitis except 4 studies.

Smoking and systemic diseases are the main confounders to induce systemic oxidative stress. Because studies with smokers and subjects that had systemic diseases in both groups were excluded during study selection procedure, all included studies had good comparability of cases and controls and were given two stars.

For exposure, oxidative biomarkers levels in case and control groups were all determined in laboratory and had secure record. Response rate was the same for both groups. So all included studies were given 3 stars.

### 3.3. Oxidative Biomarkers in the Meta-Analysis

**Figure 2:**
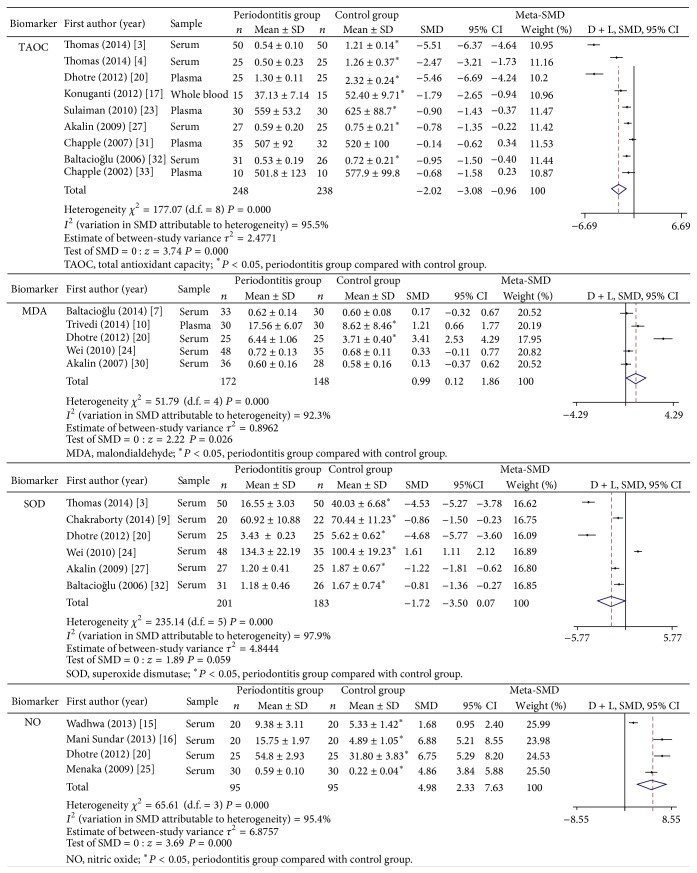
Meta-analysis of oxidative stress biomarkers in peripheral blood of periodontitis patients and controls.

Meta-analyses of four oxidative biomarkers including total antioxidant capacity (TAOC), malondialdehyde (MDA), superoxide dismutase (SOD), and nitric oxide (NO) were performed with random-effects model. Significant heterogeneity existed among studies (all *P* < 0.001 and *I* ^2^ > 90%). The results were presented in Figure 2.

In total, TAOC was determined in 9 studies. 7 studies showed lower TAOC levels in periodontitis group than in periodontal health group, and the other two showed a nonsignificant difference between two groups. Meta-analysis results show that periodontitis patients had lower TAOC levels than periodontal healthy controls (SMD = −2.02; 95% CI: −3.08, −0.96; *P* = 0.000).

MDA was determined in 5 studies; 2 studies showed higher levels in periodontitis group than in periodontal health group, and the other three showed a nonsignificant difference between two groups. Meta-analysis results show that periodontitis patients had higher MDA levels than periodontal healthy controls (SMD = 0.99; 95% CI: 0.12, 1.86; *P* = 0.026).

Of 6 articles that reported SOD, 5 showed lower SOD levels in periodontitis group than in periodontal health group, and the other one showed an opposite significant difference. The final meta-analysis results show nonsignificant difference between two groups (SMD = −1.72; 95% CI: −3.50, 0.07; *P* = 0.059).

All the 4 studies that determined NO showed higher NO levels in periodontitis group than in periodontal health group, and meta-analysis shows the same results (SMD = 4.98; 95% CI: 2.33, 7.63; *P* = 0.000).

## 4. Discussion

This meta-analysis systematically summarized the results of 16 independent studies from different countries and suggested that oxidative stress biomarkers TAOC levels in peripheral blood were lower and MDA and NO levels in peripheral blood were higher in periodontitis patients than healthy subjects, which indicated an elevation in systemic oxidative stress status in periodontitis patients. However, SOD levels in peripheral blood were not significantly different between periodontitis patients and healthy subjects. Taken together, these results clearly show that chronic periodontitis is significantly related to some but not all markers of oxidative stress status of systemic circulation.

There were no well-validated biomarkers of oxidative stress and various biomarkers were used in different studies. Antioxidants, enzymes, and the oxidation products of protein, lipids, and DNA were widely used to indicate the oxidative status and also acted as oxidative stress biomarkers. Most of biomarkers were only measured in one or two studies of the 31 included relevant articles. Our meta-analysis focused on biomarkers that were measured and reported at least in three studies, which ensure enough statistical power of meta-analysis. Systemic diseases were associated with ROS [9, 10, 15, 16], and cigarette can produce thousands of kinds of ROS molecules [17], so studies with patients of systemic disease or smokers were excluded.

Our results showed that TAOC levels decreased and MDA and NO levels increased in the peripheral blood of chronic periodontitis patients. MDA is the principal product of polyunsaturated fatty acid peroxidation that can indicate the increase of oxidative stress [18]. NO is a short-lived reactive free radical and is synthesized by the oxidative process of the guanidine of the amino acid L-arginine [19]. These suggested that periodontal inflammation might trigger systemic oxidative stress. PMN activation in peripheral blood could result in the increment of ROS in circulation. Matthews et al. demonstrated that peripheral neutrophils (a subgroup of PMN) from chronic periodontitis patients showed an increase of extracellular ROS release in vitro without exogenous stimulation [20]. Dias et al. found that proinflammatory cytokines such as IL-8, GM-CSF, and IFN-*α* increased in the plasma of periodontitis patients. These cytokines in periodontitis patients were more effective in stimulating neutrophil superoxide production than those in healthy controls [21]. Periodontitis may cause oxidative damage to multiple distant organs. An animal research showed that excessive production of lipid peroxide following periodontal inflammation was involved in oxidative DNA damage of the brain, heart, liver, and kidney in rats [22].

SOD is an enzyme that catalyses the dismutation of O_2_^•−^ (a ROS molecular) to H_2_O_2_ and O_2_ [23]. Although five studies showed lower SOD levels in periodontitis group than in periodontal health group and the other one showed an opposite significant difference, all these studies suggested the same conclusion that periodontitis could alter systemic SOD levels. When SOD levels were found lower in periodontitis patients, it can be explained as more superoxides were induced as periodontal inflammation. More SOD would be consumed and cause SOD level decrease. On the other hand, when SOD levels were found higher in periodontitis patients, it can be explained as that more SOD were produced to afford biological protection against increased superoxide generation during periodontal inflammation. However, our meta-analysis result of six studies showed that SOD levels had no significant difference between periodontitis group and healthy subjects.

This meta-analysis provides overall evidence on the relation between available biomarkers of oxidative stress and chronic periodontitis from human peripheral blood samples. However, there are some limitations caused by study heterogeneity, such as designs, sample sizes, and methods of biomarker measurement. There was a remarkable variability among selected studies regarding the types of oxidative stress biomarkers. In total, more than a dozen different biomarkers were detected in all these selected studies. All biomarkers conducted in meta-analysis were only detected in four to nine studies. Less studies numbers and small sample size may reduce the statistical power of analysis. On the another hand, different measurement methods were used in different studies even for the same oxidative stress biomarker, so that the biomarker levels were in different orders of magnitude or have different units in different studies. Effect scale SMD was used in this meta-analysis as its advantages could reduce this discrepancy. Random-effects model was applied for SMD estimation of biomarkers with high heterogeneity (all *I* ^2^ > 90%) among studies. The heterogeneity may also be induced by different definition of chronic periodontitis, different study populations (sex and age), and different biological specimens (serum, plasma, or whole blood) among different studies. Different study designs (cross-sectional, case control, or interventional study) may also produce the heterogeneity. In addition, inherent limitations of the cross-sectional and case control study designs do not allow inferences about causality concerning the association.

## 5. Conclusion

Our meta-analysis results suggested that oxidative biomarkers TAOC, MDA, and NO levels from peripheral blood samples were significantly different between periodontitis patients and healthy subjects. This evidence suggested that chronic periodontitis was associated with systemic oxidative stress in human bodies. Our findings further indicated that clinical intervention of periodontitis may be beneficial for periodontitis patients' systemic oxidative stress control and reduce its potential effect to systemic diseases. Prospective studies and randomized trials are needed to verify this hypothesis in the future.

## Supplementary Material

The methodological qualities of all the included non-randomized studies were assessed by NOS. These studies had middle or high quality. 7 studies were given 6 stars, 20 studies were given 7 stars, and 4 studies were given 8 stars.

## Figures and Tables

**Table 1:** Characteristics of the included studies.

First author (Year)	Country	Research type	Sample size		Age (years)		Periodontal parameters (mm)		Oxidative stress biomarkers
			(males/females)		(mean ± SD or mean, min–max)		(mean ± SD or median, min–max or 25%–75%)		
			Case	Control	Case	Control	Case	Control	
Thomas (2014) [24]	India	Intervention study	50 (N/A)	50 (N/A)	35–65	35–65	N/A	N/A	Serum: TAOC, SOD
Thomas (2014) [25]	India	Intervention study	25 (N/A)	25 (N/A)	N/A	N/A	N/A	N/A	Serum: TAOC, catalase
Singh (2014) [26]	India	Cross-sectional	38 (8/30)	22 (6/16)	37.5, 17–58	27.5, 22–50	PD: 3.61 (2.31–4.96) CAL: 3.99 (1.66–6.79)	PD: 1.19 (0.17–2.18) CAL: 0.15 (0.04–1.44)	Serum: SOD
Baltacio$\overset{ˇ}{\text{g}}$lu(2014) [27]	Turkey	Case control	30 (14/16)	28 (13/15)	32.70 ± 5.16	28.14 ± 3.96	PD: 3.60 (3–4.30) CAL: 3.88 (3.38–4.30)	PD: 1 (0.25–1.50) CAL: 1.25 (0.50–1.75)	Serum: TOS
Baltacıoğlu (2014) [28]	Turkey	Case control	33 (16/17)	30 (16/14)	32.55 ± 5.32	30.10 ± 4.06	PD: 4.03 (3.79–4.19) CAL: 1.4 (1.15–1.65)	PD: 1 (0.5–1.2) CAL: 0.75 (0.5–1.31)	Serum: MDA, TOS, TAOC, OSI
Chaudhary (2014) [29]	India	Intervention study	15 (9/6)	15 (7/8)	35.6 ± 5.79	32.8 ± 6.38	PD: 2.59 ± 0.23 CAL: 2.69 ± 0.22	PD: 1.53 ± 0.21 CAL: 1.45 ± 0.19	Plasma: ROM
Chakraborty (2014) [30]	India	Case control	20 (0/20)	22 (0/22)	35.90 ± 4.14	33.13 ± 6.38	PD: 3.30 ± 0.63 CAL: 3.93 ± 1.01	PD: 1.22 ± 0.63 CAL: 0.23 ± 0.32	Serum: SOD
Trivedi (2014) [14]	India	Case control	30 (11/19)	30 (6/24)	N/A	N/A	PD: 4.16 ± 0.47 CAL: 4.73 ± 0.55	PD: 1.77 ± 0.21 CAL: 1.82 ± 0.21	Plasma: MDA
Pradeep (2013) [31]	India	Case control	15 (N/A)	10 (N/A)	35.80 ± 5.93	28.20 ± 4.31	PD: 6.93 ± 1.48 CAL: 6.13 ± 1.18	PD: 1.70 ± 0.48 CAL: N/A	Serum: HNE
Thomas (2013) [32]	India	Case control	50 (N/A)	50 (N/A)	N/A	N/A	N/A	N/A	Serum: glutathione, catalase, selenium
Akpinar (2013) [33]	Turkey	Intervention study	15 (7/8)	10 (5/5)	37.7 ± 5.9	37.0 ± 7.4	PD: 5 (3–5) CAL: 8 (7–10)	PD: 1 (1-2) CAL: 0	Serum: TAS, TOS
Sezer (2013) [34]	Turkey	Cross-sectional	20 (6/14)	20 (6/14)	45.50 ± 7.50	40.75 ± 10.26	PD: 3.42 ± 0.43 CAL: 3.69 ± 0.49	PD: 2.18 ± 0.90 CAL: 0.32 ± 0.23	Serum: TAS, TOS, ARE, CRL, LOOH, prolidase, OSI
Wadhwa (2013) [35]	India	Case control	20 (N/A)	20 (N/A)	N/A	N/A	PD: 4.35 ± 0.56 CAL: 6.51 ± 0.40	PD: 0.84 ± 0.21 CAL: N/A	Serum: NO
Mani Sundar(2013) [36]	India	Cross-sectional	20 (N/A)	20 (N/A)	25–55	25–55	N/A	N/A	Serum: NO
Konuganti (2012) [37]	India	Case control	15 (N/A)	15 (N/A)	N/A	N/A	N/A	N/A	Whole blood: TAOC
Patel (2012) [38]	India	Intervention study	10 (5/5)	10 (5/5)	35.10 ± 2.51	35.10 ± 2.02	PD: 6.1 CAL: 4.1	PD: 1.3 CAL: 0	Serum: GPx
Esen (2012) [39]	Turkey	Case control	20 (4/16)	20 (4/16)	42.85 ± 9.6	40.05 ± 9.8	PD: 6.17 (5.33–6.50) CAL: 6.33 (5.67–7.09)	PD: 1.92 (1.67–2.33) CAL: 0.02 (0.01-0.02)	Serum: TAS, TOS, OSI
Dhotre (2012) [40]	India	Case control	25 (N/A)	25 (N/A)	(N/A)	(N/A)	N/A	N/A	Serum: MDA, NO, SOD, GPx Plasma: TAOC
Tamaki (2011) [41]	Japan	Intervention study	22 (10/12)	22 (10/12)	44.0 ± 19.2	43.9 ± 20.0	PD: 2.1 (1.7–2.9) CAL: 2.3 (1.8–3.0)	PD: 1.8 (1.6–1.9) CAL: 1.8 (1.6–1.9)	Plasma: oxLDL, ROM, oxidative-index
Thomas (2010) [42]	India	Case control	20 (N/A)	20 (N/A)	N/A	N/A	N/A	N/A	Serum: vitamin C
Sulaiman(2010) [12]	Syria	Intervention study	30 (9/21)	30 (9/21)	41, 23–65	34, 25–59	PD: 3.43 ± 0.45 CAL: 3.52 ± 0.44	N/A	Plasma: TAOC
Wei (2010) [13]	China	Intervention study	48 (27/21)	35 (19/16)	40.1 ± 7.3	42.1 ± 7.7	PD: 3.81 ± 0.44 CAL: 4.65 ± 0.91	PD: 1.21 ± 0.23 CAL: 0.49 ± 0.33	Serum: MDA, TOS, SOD
Menaka (2009) [43]	India	Case control	30	30	N/A	N/A	N/A	N/A	Serum: NO
Tamaki (2009) [44]	Japan	Intervention study	19 (7/12)	19 (7/12)	46.8 ± 19.1	45.3 ± 20.7	PD: 2.3 ± 0.7 CAL: 2.5 ± 0.9	PD: 1.7 ± 0.3 CAL: N/A	Plasma: ROM
Akalin (2009) [45]	Turkey	Case control	27 (0/27)	25 (0/27)	29.3 ± 3.94	29.73 ± 3.71	PD: 3.19 ± 0.16 CAL: 3.36 ± 0.25	PD: 1.25 ± 0.18 CAL: 1.49 ± 0.2	Serum: TAOC, SOD
Baltacioğlu (2008) [46]	Turkey	Case control	33 (17/16)	24 (11/13)	40.5 ± 5.5	39.3 ± 5.7	PD: 4 (3–5.3) CAL: 4.2 (2.5–7.2)	PD: 1.25 (0.5–1.9) CAL: 0.3 (0–0.8)	Serum: protein carbonyl
Konopka (2007) [47]	Poland	Case control	30 (15/15)	25 (10/15)	44.9, 35–55	33.2, 22–50	PD: 4.02 ± 0.84 CAL: N/A	PD: 1.94 ± 0.21 CAL: N/A	Serum: 8-OHdG, TAS
Akalin (2007) [48]	Turkey	Case control	36 (19/17)	28 (13/15)	40.66 ± 5.31	38.5 ± 6.10	PD: 3.92 ± 0.52 CAL: 4.45 ± 0.86	PD: 1.18 ± 0.38 CAL: 0.27 ± 0.25	Serum: MDA, TOS
Chapple (2007) [49]	UK	Intervention study	35 (12/23)	32 (N/A)	N/A	N/A	PD: 3.6 ± 0.5 CAL: N/A	PD: N/A CAL: N/A	Plasma: TAOC
Baltacıoğlu(2006) [50]	Turkey	Case control	31 (0/31)	26 (0/31)	37.4 ± 5.4	37.1 ± 4.2	PD: 3.56 ± 0.45 CAL: 3.82 ± 0.54	PD: 1.41 ± 0.25 CAL: 1.61 ± 0.27	Serum: TAOC, SOD
Chapple (2002) [51]	UK	Case control	10 (5/5)	10 (5/5)	46.1	46.9	PD: 2.9 ± 0.59 CAL: N/A	PD: N/A CAL: N/A	Plasma: TAOC

PD: pocket depth or probing depth; CAL: clinical attachment level; TAOC: total antioxidant capacity; SOD: superoxide dismutase; TOS: total oxidant status; MDA: malondialdehyde; OSI: oxidative stress index; NO: nitric oxide; ROM: reactive oxygen metabolites; HNE: 4-Hydroxy-2-nonenal; TAS: total antioxidant status; ARE: arylesterase; CRL: ceruloplasmin; LOOH: lipid hydroperoxides; GPx: glutathione peroxidase; oxLDL: oxidized low-density lipoprotein; 8-OHdG: 8-hydroxy-deoxyguanosine; N/A: not available.
