# Supplementary material for: Systemic Oxidative Stress Biomarkers in Chronic Periodontitis: A Meta-Analysis
Source: Dis Markers. 2014 Nov 16;2014:931083. doi: 10.1155/2014/931083 (PMC4247950; doi:10.1155/2014/931083)
Supplement: Supplementary file 1 — The methodological qualities of all the included non-randomized studies were assessed by NOS. These studies had middle or high quality. 7 studies were given 6 stars, 20 studies were given 7 stars, and 4 studies were given 8 stars. [file 931083.f1.doc]

**Supplemental table 1.**

**Quality assessment** of included studies with NOS*

| **First author** | **SELECTION** | **COMPARABILITY** | **EXPOSURE** | **Total** |
| --- | --- | --- | --- | --- |
|  | 1) Is the Case Definition Adequate  2) Representativeness of the Cases  3) Selection of Controls  4) Definition of Controls | 1) Comparability of cases and controls on the basis of the design or analysis | 1) Ascertainment of Exposure  2) Same method of ascertainment for cases and controls  3) Non-Response rate |  |
| Thomas B [15] | ★★★ | ★★ | ★★★ | 8 |
| Thomas B [16] | ★★ | ★★ | ★★★ | 7 |
| Singh N [17] | ★★ | ★★ | ★★★ | 7 |
| Baltacıoğlu E [18] | ★★ | ★★ | ★★★ | 7 |
| Baltacıoğlu E [19] | ★★★ | ★★ | ★★★ | 8 |
| Chaudhary S [20] | ★★ | ★★ | ★★★ | 7 |
| Chakraborty S [21] | ★★ | ★★ | ★★★ | 7 |
| Trivedi S [14] | ★★ | ★★ | ★★★ | 7 |
| Pradeep AR [22] | ★★★ | ★★ | ★★★ | 8 |
| Thomas B [23] | ★ | ★★ | ★★★ | 6 |
| Akpinar A [24] | ★★ | ★★ | ★★★ | 7 |
| Sezer U [25] | ★★ | ★★ | ★★★ | 7 |
| Wadhwa D [26] | ★★ | ★★ | ★★★ | 7 |
| Sundar NM [27] | ★★ | ★★ | ★★★ | 7 |
| Konuganti K [28] | ★ | ★★ | ★★★ | 6 |
| Patel SP [29] | ★★ | ★★ | ★★★ | 7 |
| Esen C [30] | ★★ | ★★ | ★★★ | 7 |
| Dhotre PS [31] | ★ | ★★ | ★★★ | 6 |
| Tamaki N [32] | ★★ | ★★ | ★★★ | 7 |
| Thomas B [33] | ★ | ★★ | ★★★ | 6 |
| Abou S AE [12] | ★★ | ★★ | ★★★ | 7 |
| Wei D [13] | ★★ | ★★ | ★★★ | 7 |
| Menaka KB [34] | ★ | ★★ | ★★★ | 6 |
| Tamaki N [35] | ★★ | ★★ | ★★★ | 7 |
| Akalin FA [36] | ★★ | ★★ | ★★★ | 7 |
| Baltacioğlu E [37] | ★★★ | ★★ | ★★★ | 8 |
| Konopka T [38] | ★ | ★★ | ★★★ | 6 |
| Akalin FA [39] | ★★ | ★★ | ★★★ | 7 |
| Chapple IL [40] | ★★ | ★★ | ★★★ | 7 |
| Baltacioğlu E [41] | ★★ | ★★ | ★★★ | 7 |
| Chapple IL [42] | ★ | ★★ | ★★★ | 6 |

*NOS: Newcastle-Ottawa Quality Assessment Scale
